# One-year impact of a multicomponent, street-level design intervention in Mexico City on pedestrian crashes: a quasi-experimental study

**DOI:** 10.1136/jech-2022-219335

**Published:** 2022-12-19

**Authors:** Luz Mery Cárdenas-Cárdenas, Tonatiuh Barrientos Gutiérrez, D Alex Quistberg, Luis Chias-Becerril, Armando Martínez-Santiago, Héctor Reséndiz Lopez, Carolina Perez Ferrer

**Affiliations:** 1 Center for Population Health Research, National Institute of Public Health, Cuernavaca, Morelos, Mexico; 2 Urban Health Collaborative, Drexel University, Philadelphia, Pennsylvania, USA; 3 Institute of Geography, National Autonomous University of Mexico, Ciudad de Mexico, Ciudad de México, Mexico; 4 CONACYT, National Institute of Public Health, Cuernavaca, Morelos, Mexico

**Keywords:** ACCIDENTS, PUBLIC HEALTH, WOUNDS AND INJURIES, DEATH

## Abstract

**Background:**

Mexico City implemented the *Pasos Seguros* programme to prevent pedestrian injuries and deaths at dangerous road intersections, which included street-level design changes, such as visible pedestrian crossings, sidewalk widening, refuge islands, lane reductions, pedestrian signals and adjustment of traffic light timing at these intersections. Few studies in low and middle-income countries (LMICs) have evaluated the effect of such interventions on pedestrian safety.

**Aim:**

Assess the effectiveness of the *Pasos Seguros* programme at reducing total, injury and fatal pedestrian-motor vehicle crashes.

**Methods:**

Two-group quasi-experimental design. Monthly pedestrian crashes were obtained from the road incident database from Mexico City’s Citizen Contact Center. The programme’s effectiveness was evaluated by comparing 12 months preintervention to 12 months postintervention implementation using a negative binomial regression with random intercept with a difference-in-difference estimation. A qualitative comparative analysis was used to find the configuration of intersection characteristics and programme components associated with a decrease in pedestrian crashes.

**Results:**

Total pedestrian crashes were reduced by 21% (RR 0.79; 95% CI 0.62 to 0.99) after implementation of *Pasos Seguros* programme. This reduction was observed for pedestrian injury crashes (RR 0.79; 95% CI 0.62 to 1.00) and for fatal crashes (RR 0.61; 95% CI 0.13 to 2.92) although not statistically significant for the latter. A decrease in pedestrian crashes was found at the most complex intersections where more of the programme components was implemented.

**Conclusion:**

The *Pasos Seguros* programme successfully decreased total and injury pedestrian crashes. Similar interventions may improve walking safety in other LMIC cities.

**WHAT IS ALREADY KNOWN ON THIS TOPIC**

- The frequency of road traffic collisions is disproportionately higher in urban areas from developing countries; pedestrians are highly affected especially at intersections.
- Mexico City’s government implemented the *Pasos Seguros* programme, a multicomponent, street-level design intervention, to make intersections safer for pedestrians.
- The interventions to reduce road accidents in low and middle-income countries have been directed at drivers and vehicle occupants but not at the geometric design improvement of intersections to reduce pedestrian risk.

**WHAT THIS STUDY ADDS**

- Street-level geometric design improvements and adding traffic lights and pedestrian signal traffic control devices at intersections led to a reduction in total and injury pedestrian crashes.
- The multicomponent intervention programmes are more effective in the most complex intersections (with four or more legs and two directions of traffic in the main road).

**HOW THIS STUDY MIGHT AFFECT RESEARCH, PRACTICE OR POLICY**

- The results of this study encourage the maintenance of intervened intersections and expansion of the programme to other intersections in Mexico City and potentially other cities in low and middle-income countries.
- The evaluation of the effectiveness of road interventions oriented to their geometric redesign is necessary to make the case for public investments in road infrastructure.

## Background

Globally, road traffic accidents are the eighth leading cause of death for all age groups and the main cause of death in children and young people between 5 and 29 years.^1^ The frequency of road traffic collisions is disproportionately higher in developing countries, with urban areas being the most affected.^1^ In Mexico City, the second-most populous city in Latin America and the Caribbean,^2^ there were 367 deaths and 3151 injuries from road traffic collisions in 2019.^3^ Pedestrians represented 45% and 21% of deaths and injuries, respectively, which is higher than that reported for the Americas region.^1 4^

In Mexico City, most pedestrian crashes occur at intersections that are considered crash hotspots.^5^ From 2015 to 2017, Mexico City implemented the *Pasos Seguros* programme, a citywide programme targeting intersections between arterials and other primary roads, located in 12 road corridors, where 76% of road incidents take place.^5^ The objective of this programme was to make intersections identified as crash hotspots safer for pedestrians.^5^ The programme was implemented in three stages, starting with the most dangerous intersections. The first in 2015 in which 56 intersections were intervened, the second in 2016 with the intervention of 40 intersections and the third in 2017 with the intervention of 10 intersections. One or more components of the programme were implemented. These were: street-level geometric design improvements (eg, improving and enabling pedestrian protection zones such as sidewalks and refuge islands; pavement markings to pedestrian crossings, bicycle waiting and traffic direction arrows), adjustment of traffic signal timing and adding pedestrian signal traffic control devices.^5^ The programme was implemented with an awareness campaign directed at pedestrians and motorists.

These interventions can help protect pedestrians by reducing the amount of time they spend in the roadway by reducing crossing distances, providing more dedicated walking space along roadways, making pedestrians more visible to drivers and reducing motor vehicle speeds.^6–12^ The presence of pedestrian refuge islands, a smaller number of lanes and widening of sidewalks has been associated with lower risk of pedestrian exposure to vehicle traffic and fewer pedestrian crashes at intersections.^6–8^ Likewise, evaluation studies of multicomponent geometric design improvement and vehicle flow reduction interventions implemented in cities in developed countries show a significant decrease in the number of pedestrian crashes.^10–12^ The effectiveness of these interventions could be influenced by the built environment, the characteristics of the roads, motor vehicle and pedestrian traffic and the combination of interventions implemented.^9 12 13^

In low and middle-income countries, specifically in Latin America, most interventions to reduce road accidents have been directed at drivers and vehicle occupants, but not at the geometric design improvement of roads to reduce pedestrian risk.^14 15^ The *Pasos Seguros* programme was, therefore, innovative because it tackled the urban infrastructure around intersections. The main aim of this study was to assess whether the *Pasos Seguros* programme was associated with a decrease in total pedestrian crashes and pedestrian crashes resulting in injuries or deaths. As secondary aim, we looked to identify the configurations of characteristics of the intersections (number of legs and number of directions of traffic in the main road) and components of the *Pasos Seguros* programme (visible pedestrian crossings, sidewalk widening, refuge island, lane reduction, traffic lights and pedestrian signals), which were associated with the reduction in pedestrian crashes.

## Materials and methods

### Study design and data

A two-group quasi-experimental design was used to compare total pedestrian crashes and pedestrian crashes, resulting in injuries or deaths before and after the implementation of the *Pasos Seguros* programme. *The Pasos Seguros* intersections were georeferenced from a list of addresses requested from the Citizen Security Ministry (Secretaría de Seguridad Ciudadana in Spanish) of Mexico City. These intersections were validated using Google Street View (GSV). We included 91 *Pasos Seguros* intersections that were more than 80 m from another *Paso Seguro* intersection to avoid pedestrian crashes being included in more than one intersection (online supplemental Appendix 1). The exact date of the intervention of each intersection was not recorded in administrative records and it was not possible to recover it via local authorities; thus, to estimate the date of intervention, we used GSV historical imagery to identify for each *Paso Seguro* intersection the last date of the location without any intervention, the first date of the location with intervention. The number of pictures available in the historical imagery varied by intersection, 67% of *Pasos Seguros* had a difference of less than or equal to 6 months between the last date of the location without any intervention and the first date of the location with intervention. A difference of 7 to 12 months was found for 24% of intersections, and 8% had a difference greater than 1 year.

10.1136/jech-2022-219335.supp1 Supplementary data

Monthly pedestrian crashes, during 12 months before and after the last date without intervention and the first date with the intervention, were taken as the before and after period, respectively. Each *Paso Seguro* intersection had a different date to define the before and after period. We also used GSV to identify the components of the *Pasos Seguros* programme implemented at each location and intersection (online supplemental Appendix 2). We evaluated inter-rater reliability of the GSV assessment in a subsample of 14% of intersections (online supplemental Appendix 3). This study included the characteristics and components with a percent agreement greater than 80% or Kappa coefficient equal or greater than 0.3, specifically: number of legs, number of traffic directions on the main road, visible pedestrian crossing, sidewalk widening, refuge island, lane reduction, traffic lights and pedestrian signals.

### Control intersection selection

As a control group, non-intervened intersections were selected. A geographic data set of non-intervened intersections was generated. All possible control intersections of the road network were located, then we identified 263 neighbouring non-intervened intersections (with a distance ≥100 to <800 metres from intervened intersections), which were on the same road as the *Pasos Seguros* intersections. We dropped 66 non-intervened intersections that met the distance criteria but were adjacent to the *Pasos Seguros* intersection to reduce spillover effects as have been done in other studies.^9 11^ Non-intervened intersections also were more than 80 m apart from each other and had the same speed limit as *Pasos Seguros* (50 km/hour). We used a Propensity Score Matching with a level=0.001, with one-to-one nearest neighbour matching without replacement to select two control intersection for each intervened intersection (online supplemental Appendix 1).^16^ The observable characteristics that were used to estimate the probability of participation included characteristics of the environment, roads and intersections that according to the evidence are associated with pedestrian and vehicle volume.^6 7 17 18^ Environmental characteristics of each location’s neighbourhood were publicly available,^19–21^ these included: proportion of employed population, proportion over 65 years old, proportion under 18 years old, number of educational establishments, number of hospitals and presence of at least one Metro (subway) station in a 1 km aerial radius. The characteristics of roads and intersections included the hierarchical classification of roads that converge at the intersection (intersection of two principal arterial roads or intersection of one principal arterial roadway and major or minor collector roadways). The before and after period for each control intersection was assigned according to the dates of the *Paso Seguro* intersection of which it was controlled.

### Outcome variables

Pedestrian crashes included any road traffic incident involving a pedestrian struck by a motor vehicle. The data on pedestrian crashes were obtained from the road incident database from the Mexico City Command, Control, Computing, Communications and Citizen Contact Centre (C5).^22^ The C5 database contains geo-referenced traffic crashes reported through emergency tools such as calls to 911, mobile 911 App, video surveillance cameras and emergency call buttons located in streets and public areas.^22^ C5 database is freely available and has data from 2014 to 2020.^22^ The earliest data used in this study were February 2014 and the latest data were February 2020. We used incident data with codes ‘injured-pedestrian crash’ or ‘corpse-pedestrian crash’, which included pedestrian crashes, resulting in injuries or deaths and that were confirmed by an emergency unit at the scene. Duplicate records were dropped in two stages. First, we dropped the records with duplication in all variables, except the serial/identification number variable. Second, records were considered duplicates if they shared the same incident code, geographic coordinates and the reports were 1 hour or less apart.^23^ We calculated the total number of pedestrian crashes per month at the intersection level as the sum of the incidents coded as injured-pedestrian crash and those coded as corpse-pedestrian crash. For intersections of two principal arterial roads, we included pedestrian crashes whose straight-line distances were ≤80 m from the intersection’s geographic coordinates. We used a distance ≤50 m in intersections of one principal arterial roadway and major or minor collector roadways. These distances agree with previous studies and are according to the size of the roadway.^9 17 18^ For the outcome, we use the count of pedestrian crashes and also, we calculated the rate of total of pedestrian crashes, pedestrian injury crashes and fatal pedestrian crashes per 100 000 inhabitants per square kilometre by intersection. We used the population density in the neighbourhood, defined as the basic geostatistical area—AGEB in Spanish—where the intersection was located, as the denominator.

### Analysis

In the descriptive analysis, we calculate, by intersection, the mean of pedestrian crashes before and after the *Pasos Seguros* programme implementation and compare them using a paired t test. Central tendency and dispersion measures were used in continuous variables and frequencies and percentages in categorical variables. To assess the effectiveness of the *Pasos Seguros* programme, we used negative binomial regression models with a random intercept for intersections and road corridors to consider repeated measures at the intersection level and the nested intersections in the road corridors. The regression models were adjusted by the population density at the AGEB in which the intersection is placed. The models estimate risk ratios and 95% CI. We used a difference-in-difference (DiD) approach to estimate the effectiveness of the programme in which an interaction term between the group variable (intervened vs non-intervened) and the period (before vs after) was included in the models. This interaction term assessed whether the changes in the monthly number of pedestrian crashes from the before to the after period was different between *Pasos Seguros* and control intersections. We used a marginal postestimation to estimate the absolute effectiveness of the programme. The parallel tendency assumption was fulfilled (online supplemental Appendix 4).

We used a qualitative comparative analysis (QCA) to identify the configurations of components of the *Pasos Seguros* package and characteristics of the intersections associated with the reduction in pedestrian crashes.^24^ The QCA combines qualitative and quantitative techniques and is useful to study small samples.^24^ QCA is a case-oriented method that uses the set theory and Boolean algebra to identify patterns or configurations (combinations of attributes), rather than single conditions or variables, associated with an outcome.^24–26^ QCA uses two goodness-of-fit statistics. The consistency assesses the degree to which the cases sharing a given condition or combination of conditions, and the coverage that means the proportion of cases covered in a specific configuration.^24^

The QCA was done only with the *Pasos Seguros* intersections. For this analysis, the outcome variable was the difference in pedestrian crashes comparing the mean of pedestrian crashes before the implementation of the *Pasos Seguros* programme with the same mean after programme implementation. This difference was calibrated using the standardisation method to convert their values into an interval between 0 and 1.^24^ Intersection characteristics and programme components were dichotomous variables for which an uppercase letter was assigned for the presence of the attribute and a lowercase letter in the absence of the attribute. The resulting configurations had to pass two tests: first, having a consistency greater than 0.7, and second, having a consistency greater than non-consistency.^27^

As a sensitivity analysis, we analysed all collisions to evaluate whether the *Pasos Seguros* programme made these intersections safer for all road users. All collisions included vehicle-to-vehicle collisions resulting in fatal, injuries or without injuries; traffic accidents involving cyclists, motorcyclists and scooters; pedestrian crashes and other traffic accidents that included car overturned and collisions with objects.

Stata V.14.0 (STATA Corporation, College Station, Texas) and R i386 V.3.5.1 were used. An α<0.05 was statistically significant.

## Results

**Figure 1:**
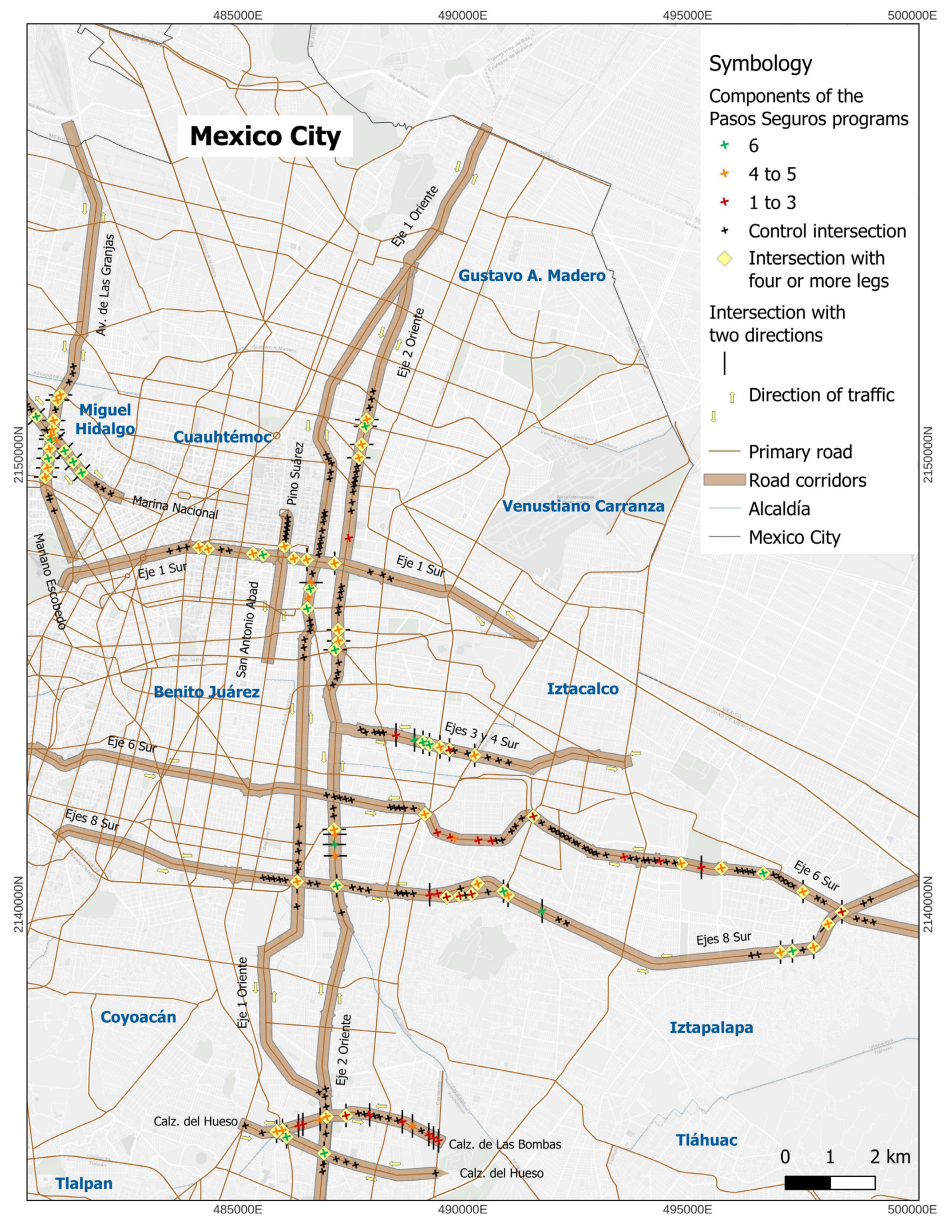
*Pasos Seguros* and control intersections. *Pasos seguros’* characteristics and components of the programme. Figure elaborated by research team.

Table 1 shows the characteristics of intersections and the frequency of the components of the *Pasos Seguros* programme implemented. 68.1% of *Pasos Seguros* intersections had four or more legs and 70.3% had two traffic directions on the main road. In all *Pasos Seguros* intersections, visible pedestrian crossings were implemented. A total of 73.6% of intersections had sidewalk widening and refuge islands. The least frequent component was traffic light installation (38.5%). Figure 1 summarises the number of components of the *Pasos Seguros* programme implemented and the characteristics of intersection.

**Table 1:** *Pasos Seguros* intersection’s characteristics and components

Characteristic or component	n (%)
***Intersections' characteristics***	
Number of legs (L) Four or more	62 (68.1)
Number of traffic directions (D) Two	64 (70.3)
***Components of the Pasos Seguros Programm***	
Visible pedestrian crossings (C)	91 (100)
Sidewalk widening (A)	67 (73.6)
Refuge islands (I)	67 (73.6)
Lane reductions (W)	62 (68.1)
Traffic lights (T)	35 (38.5)
Pedestrian signals (P)	60 (65.9)

During the study period, the total number of pedestrian crashes at intersections was 1331 (1299 were pedestrian injury crashes and 32 were pedestrian fatal crashes). Of the total pedestrian crashes, 721 (54.2%) occurred at *Pasos Seguros* intersections. As shown in table 2, *Pasos Seguros* intersections had a significantly higher rate of pedestrian crashes in the before period with a mean of 6.88 (SD=28.04) total pedestrian crashes per intersection in comparison with control intersections, in which the mean rate was 1.50 (SD=2.94). Similar results were found for pedestrian injury crashes (table 2). Comparing the before and after period, there was a decrease in all types of pedestrian crashes, this reduction was grater in in the *Pasos Seguros* group (table 2).

**Table 2:** Rate of pedestrian crashes (per 100 000 people per square kilometre) in the *Pasos Seguros* group and control group*

Type of pedestrian crash	Group	Before mean (SD)	After mean (SD)	Difference mean (SD)
Total pedestrian crashes	*Pasos Seguros*	6.88 (28.04)	4.67 (18.90)	−2.20 (11.77)
	Control	1.50 (2.94)†	1.40 (3.43)	−0.10 (2.07)
Pedestrian injury crashes	*Pasos Seguros*	6.59 (25.96)	4.62 (18.90)	−1.97 (9.72)
	Control	1.47 (2.88)†	1.38 (3.41)	−0.09 (2.04)
Pedestrian fatal crashes	*Pasos Seguros*	0.29 (2.24)	0.05 (0.18)	−0.24 (2.24)
	Control	0.03 (0.24)	0.02 (0.15)	−0.01 (0.28)

*Per intersection per study period.

†P value <0.05 to compare the rate of pedestrian crashes between the *Pasos Seguros* and control group in the before period.

In regression models, pedestrian crashes were reduced on average by 21% (RR=0.79; 95% CI 0.62 to 0.99) at intersections in the *Pasos Seguros* programme, which meant an average reduction of 0.16 pedestrian crashes per intersection per month. In terms of pedestrian crashes resulting in injuries, the results were nearly identical (RR=0.79, 95% CI 0.62 to 1.00, DiD=−0.15, 95% CI −0.25 to −0.05). For fatal pedestrian crashes, a 39% decrease was observed, but it was not statistically significant (table 3).

**Table 3:** Effectiveness of the *Pasos Seguros* Programme (n=6546 months intersection)

Type of pedestrian crash	RR (95% CI)	Dif-in-Dif (95% CI)
Total pedestrian crashes	0.79 (0.62 to 0.99)*	−0.16 (-0.26 to -0.06)†
Pedestrian injury crashes	0.79 (0.62 to 1.00)	−0.15 (-0.25 to -0.05)†
Pedestrian fatal crashes	0.61 (0.13 to 2.92)	−0.00 (-0.01 to 0.004)

*p<0.05

† p<0.01

Table 4 presents the truth table resulting from the QCA analysis. The most frequent configuration of intersection characteristics and components of *Pasos Seguros* programme was LDCAIWTP (16.48%). This configuration represented intersections with four or more legs (L), two directions of traffic on the main road (D), and with all the components of the *Pasos Seguros* programme implemented, such as visible pedestrian crossings (C), sidewalk widening (A), refuge island (I), lane reduction (W), traffic lights (T), and pedestrian signals (P). The second most frequent configuration was LDCAIWtP (14.29%).

**Table 4:** Configurations of *Pasos Seguros* intersection’s characteristics and components*

Configurations	Frequency	%
LDCAIWTP	15	16.48
LDCAIWtP	13	14.29
LDCAIWtp	4	4.40
LDCAIwTP	2	2.20
LDCAiWTP	2	2.20
LDCAiWtP	2	2.20
LDCaIWtP	2	2.20
LDCaiwtp	2	2.20
LdCAIWTP	4	4.40
LdCAIWtP	5	5.49
LdCAIWtp	2	2.20
LdCaiwtp	2	2.20
lDCAIWTP	3	3.30
lDCAIWtp	3	3.30
lDCAIwtp	2	2.20
lDCaiwtp	3	3.30
ldCAiwtp	2	2.20
ldCaIwtp	4	4.40
Other†	19	24.84
Total	91	100

L, intersection with four or morelegs; D, two traffic directions in the main road; C, visible pedestrian crossings; A, sidewalk widening; I, refuge island; W, lane reduction; T, traffic lights; P, pedestrian signals.

*The upper-case letter indicates the presence and, the lower-case letter indicates the absence.

†Configurations with a frequency of 1

The configuration L*D*C*A*i*W*T*P was associated with a decrease in pedestrian crashes. In other words, the decrease in pedestrian crashes was associated with *Pasos Seguros* intersections with four or more legs (L) and two directions of traffic (D) and in which visible pedestrian crossings (C), sidewalk widening (A), lane reduction (W), traffic lights (T) and pedestrian signals were implemented (P). This configuration had an acceptable consistency (0.803, p<0.001), but a low coverage (0.035), this means that this group of intersections were more complex and got most of the components of the *Pasos Seguros* programme, but their frequency was low.

The sensitivity analysis showed consistent decrease in the total number of collisions associated with the *Pasos Seguros* programme (RR=0.86; 95% CI 0.74 to 0.98) (DiD=−1.56, 95% CI −2.56 to −0.57).

## Discussion

We found that the *Pasos Seguros* programme, designed to increase the safety of intersections previously identified as dangerous for pedestrians in Mexico City, reduced total pedestrian crashes and pedestrian injury crashes by 21%. We found similar results evaluating the effectiveness of the programme on total collisions in our sensitivity analysis.

The effectiveness found in this study was similar to the 30% reported in a study in New York City, in which 118 intervened intersections as part of the Vision Zero Policy were evaluated.^9^ Another programme in New York City has reported a 47% decrease in pedestrian crashes secondary to the implementation of high visibility crosswalks at the intersections.^11^ The results of this study have consistency with previous cross-sectional studies in which intersection characteristics such as pedestrian refuge islands, a smaller number of lanes, and widening of sidewalks have been associated with fewer pedestrian crashes.^6 7 10 13 28^

We did not find a reduction in fatal pedestrian crashes. This was probably due to low monthly numbers of fatal pedestrian crashes and the time periods examined. Fatal pedestrian crashes were a rare event that represented only 2.4% (32/1331) of total pedestrian crashes at intersections. This low frequency in fatalities could be because we used a data set containing only events confirmed at the scene, which means an under-reporting of around 53% of deaths do not occur at the accident site.^4^

This study identified the combinations of *Pasos Seguros* intersection’s characteristics and components associated with decreased pedestrian crashes. We found that pedestrian crashes decreased at the most complex intersections (with four or more legs and two directions of traffic in the main road), where most of the *Pasos Seguros* programme components were implemented (visible pedestrian crossings, sidewalk widening, lane reduction, traffic lights and pedestrian signals). It has been reported that, in comparison with only one component, the combination of refuge islands with lane reduction has been more effective in reducing pedestrian crashes.^9^ Until now, no study has evaluated combinations of a greater number of geometric redesign components and characteristics of intersections, thus the QCA used in this study represents an opportunity to understand the complexity of road safety interventions.

In the sensitivity analysis, we found that the *Pasos Seguros* programme decreased all collisions. This result is consistent with a meta-analysis of evaluation studies, which reports a reduction of 15% of injury collisions associated with traffic calming measures.^29^ According to our results, a multicomponent road intervention at the intersection level, like the one evaluated here, could make dangerous intersections safer for all road users and not only for pedestrians.

An important threat to validity in before–after studies, when the intervention is assigned to a group with higher values in the outcome, is the regression to mean effect (RTM). Individuals or groups with higher-than-average values will have measures closer to the mean on subsequent measurement.^30^ However, in this study, we use a DiD approach to compare the changes in the monthly number of pedestrian crashes during the after period between the *Pasos Seguros* and control group relative to the number of pedestrian crashes during the before period, controlling for the RTM.^27^ DiD does not require similar baseline means in treatment and control groups given that the counterfactual is the trend in the control group.^30^

This study has some limitations. First, we only had 1 year as before and after period due to the data availability. However, we used a robust design that included a before and after evaluation, with monthly pedestrian crashes, for each *Pasos Seguros* intersection and control intersections. This design allowed us to control for factors that globally affected pedestrian crashes in Mexico City. Second, the road incidents used in this study did not include mild pedestrian crashes (ie, no reported pedestrian injuries), so the effectiveness reported here is centred on pedestrian injury crashes and fatal pedestrian crashes, which were the target outcomes of the *Pasos Seguros* programme.^5^ Third, the injury and fatal crashes used in this study only represent those that were confirmed at the scene and did not include the records later updated based on medical care services. However, this is the only data set that contains georeferenced information on pedestrian crashes before the implementation of the *Pasos Seguros* programme in Mexico City. A differential report of injuries/deaths between *Pasos Seguros* and control groups or before/after is unlikely. Fourth, we did not include the pedestrian and vehicular volume at the intersections studied because this information is not available. We acknowledge that these variables could be related to both; the treatment assignment and the change in the outcome over time, so in this study, we included proxies of the pedestrian, and vehicle volume in the propensity score matching to address this confusion.^30^ Also, we included the population density at AGEB level as adjustment variable in the regression models as a proxy for pedestrian volume.^31^ Fifth, the definition of the before–after period depended on availability of images in GSV. However, this did not affect the effectiveness reported here (online supplemental Appendix 5). Seventh, the data used in this study did not allow us to explore differential effects of the intervention by age groups.

This is the first study to assess the effectiveness of a multicomponent road safety programme in the Latin-American context. In conclusion, the *Pasos Seguros* programme implemented in Mexico City decreased total pedestrian crashes and pedestrian injury crashes. This decrease was observed especially at the most complex intersections and with almost all the components of the programme implemented. This kind of multicomponent programme could be implemented to protect the pedestrians in other cities in the Mexican and Latin-American context.

## Data Availability

Data are available in a public, open access repository. Data are available upon reasonable request. [dataset] [19] Instituto Nacional de Estadística y Geografía. Censo de Población y Vivienda 2010 [Internet]. INEGI. INEGI; 2016 [cited 2022 September 26]. Available from: https://www.inegi.org.mx/programas/ccpv/2010/%23Datos_abiertosdataset] [20] Instituto Nacional de Estadística y Geografía. Directorio Estadístico Nacional de Unidades Económicas [Internet]. [cited 2022 September 26]. Available from: https://www.inegi.org.mx/app/descarga/?ti=6[dataset] [21] Gobierno de la Ciudad de México. Ubicación de líneas y estaciones del Sistema de Transporte Colectivo Metro [Internet]. [cited 2022 September 26]. Available from: https://datos.cdmx.gob.mx/dataset/lineas-y-estaciones-del-metro[dataset] [22] Gobierno del Estado de México. Incidentes viales reportados por C5 [Internet]. [cited 2020 March 30]. Available from: https://datos.cdmx.gob.mx/explore/dataset/incidentes-viales-c5/table/?disjunctive.incidente_c4The location and characteristics of the intersections can be requested from the authors.
