# Supplementary material for: One-year impact of a multicomponent, street-level design intervention in Mexico City on pedestrian crashes: a quasi-experimental study
Source: J Epidemiol Community Health. 2022 Dec 19;77(3):140–6. doi: 10.1136/jech-2022-219335 (PMC7614172; doi:10.1136/jech-2022-219335)
Supplement: Supplementary data [file jech-2022-219335supp001.pdf]

One-year impact of a multicomponent, street-level design intervention in Mexico City on pedestrian crashes: a quasi-experimental study

Appendix 1. Flow chart of the *Pasos Seguros* and control intersections which were studied*

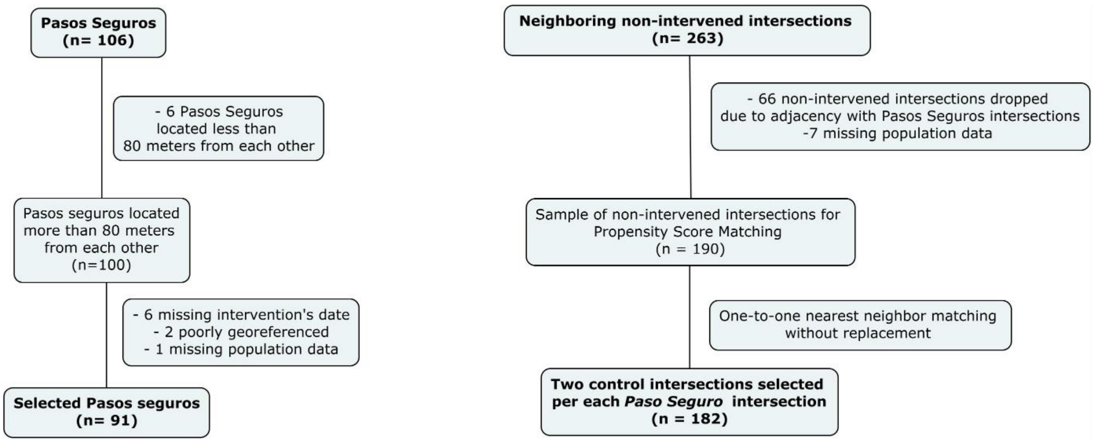

* Figure elaborated by research team

Appendix 2. Intersection characteristics and components of the *Pasos Seguros* program

**Characteristic or component**	**Definition**
Intersection characteristics	
Number of legs	Number of legs that converge at the intersection
Number of traffic directions	Number of traffic directions on the main road (one direction vs two directions)
Number of lanes in the main road	Number of lanes in the main road of the intersection
Total of lanes at the intersection	Total of lanes obtained by adding the lanes of all the roads that converge at the intersection
Components of the Pasos Seguros program	
Visible pedestrian crossings	The pedestrian crossing is painted and is visible in at least one of the legs of the intersection
Sidewalk widening	The sidewalk has one of these interventions: boundary with paint, bollards, or flowerpots.
Refuge islands	In at least one of the legs of the intersection, a refuge island was built, or the refuge island had one of these interventions: boundary with paint, bollards, or flowerpots.
Lane reduction	Decrease in the total of lanes secondary to a sidewalk expansion, or a refuge island
Traffic lights	Installation of at least one traffic light to regulate vehicular flow in at least one of the legs of the intersection
Pedestrian signals	Installation of at least one pedestrian signal in at least one of the legs of the intersection

Appendix 3. Reliability of the Google Street View assessment of the characteristics of the *Pasos Seguros* intersections and program components

Two independent observers evaluated 14% of *Pasos Seguros* intersections. We used percent agreement or Kappa coefficient to assess reliability. Results are presented in Table 1.

Table 1. **Inter-rater reliability of the Google Street View assessment of the characteristics of the program components and *Pasos Seguros* intersections**

Characteristic or component	Reliability[a]
Number of legs	0.67
Number of traffic directions	0.45
Number of lanes in the main road[b]	0.29
Total of lanes at the intersection[c]	0.22
Visible pedestrian crossings	93%
Sidewalk widening	80%
Refuge islands	0.35
Lane reduction	0.30
Traffic lights	0.41
Pedestrian signals	0.48

[a] We used percent agreement when the distribution of observations did not allow to calculate a Kappa coefficient

[b] Categorized in: ≤ 4; > 4 ≤ 8; > 8

[c] Categorized in: ≤ 10; > 10 ≤ 25; > 25

Appendix 4. Evaluation of parallel tendencies assumption

We tested the parallel tendencies assumption for the before period through a negative binomial regression model, using the time (month) as a discrete variable. An interaction term between the time and the group variable was included. This term assessed whether the changes in the number of pedestrian crashes along the time were different between *Pasos Seguros* and control intersections. As shown in the Figure 1, we did not find evidence of different tendencies before the program implementation (Slope rate = 1.01; 95% CI = 0.97 - 1.06; p = 0.541).

Figure 1. Trends of the rate of total pedestrian crashes during twelve months before intervention, by treatment group

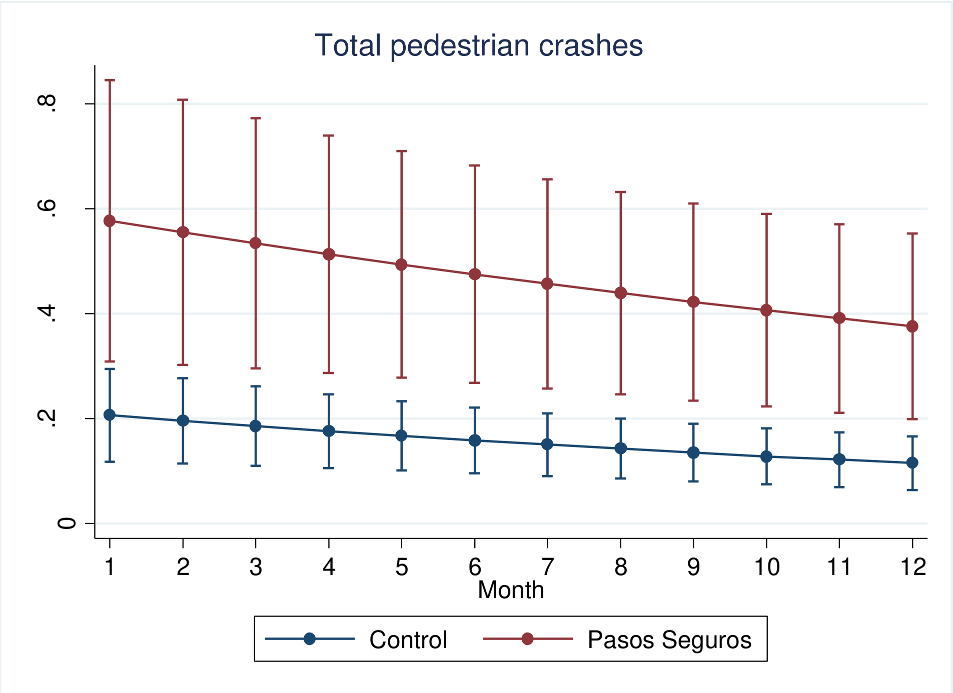

Appendix 5. Effectiveness of the *Pasos Seguros* program by date of availability of images before and after the intervention

The number of images available in the historical imagery varied by intersection, the range of the difference between the last date of the location without any intervention, and the first date of the location with intervention was 1 to 29 months with 67% of *Pasos Seguros* with a difference of less than or equal to six months.

To assess if the effectiveness observed in the study depended on the time between the two Google Street View images used to determine before and after period, we stratified the analysis in three categories of this difference (less or equal than 6 months, 7 to 12 months, and more than 12 months). Results are presented in Table 2, sections A, B, and C. The direction of the effectiveness estimator was maintained, but these were not significant. The effectiveness was greater with an increase in the time between images, but their effect on the results was not substantive because of a high percentage of intersections with the two pictures in a range less or equal to six months.

Table 2. Effectiveness of the *Pasos Seguros* program by date of availability of images before and after the intervention

A. Date difference less or equal than 6 months (n = 4,392 months intersection)

Type of pedestrian crash	RR (95% CI)	Dif-in-Dif (95% CI)
Total of pedestrian crashes	0.86 (0.65 to 1.14)	-0.17 (-0.32 to -0.03)*
Pedestrian injury crashes	0.87 (0.65 to 1.15)	-0.16 (-0.30 to -0.03)*
Pedestrian fatal crashes	0.72 (0.14 to 3.79)	-0.01 (-0.02 to 0.01)

* $p<0.05$

B. Date difference 7 to 12 months (n = 1,656 month intersection)

Type of pedestrian crash	RR (95% CI)	Dif-in-Dif (95% CI)
Total of pedestrian crashes	0.73 (0.45 to 1.19)	-0.12 (-0.26 to 0.02)
Pedestrian injury crashes	0.75 (0.46 to 1.22)	-0.11 (-0.26 to 0.03)
Pedestrian fatal crashes	NE	NE

NE: No estimable due to low frequency of fatal pedestrian crashes

C. Date difference more than 12 months (n = 498 months intersection)

Type of pedestrian crash	RR (95% CI)	Dif-in-Dif (95% CI)
Total of pedestrian crashes	0.29 (0.09 to 0.93)*	-0.21 (-0.44 to 0.02)
Pedestrian injury crashes	0.28 (0.09 to 0.88)*	0.22 (-0.45 to 0.01)
Pedestrian fatal crashes	NE	NE

[*] p<0.05; [**] p<0.01

NE: No estimable due to low frequency of fatal pedestrian crashes
